# Supplementary material for: The causal role of circulating inflammatory markers in osteoporosis: a bidirectional Mendelian randomized study
Source: Front Immunol. 2024 Jul 18;15:1412298. doi: 10.3389/fimmu.2024.1412298 (PMC11291241; doi:10.3389/fimmu.2024.1412298)
Supplement: Supplementary file 1 [file DataSheet_1.zip › supplementary material/CIM-OSTEOPOROSIS /GCST90274819.csv_forest.pdf]

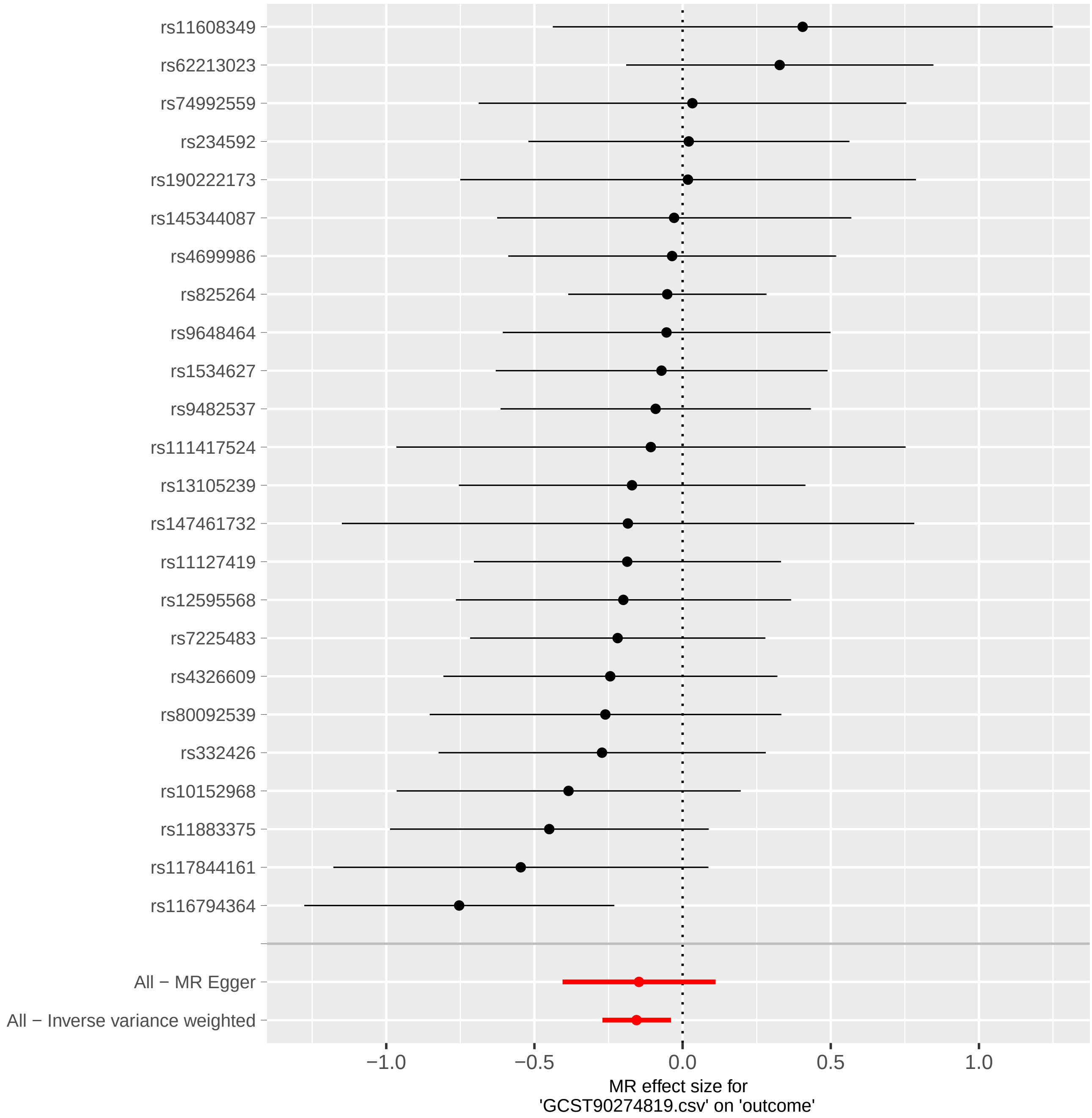
rs11608349
rs62213023
rs74992559
rs234592
rs190222173
rs145344087
rs4699986
rs825264
rs9648464
rs1534627
rs9482537
rs111417524
rs13105239
rs147461732
rs11127419
rs12595568
rs7225483
rs4326609
rs80092539
rs332426
rs10152968
rs11883375
rs117844161
rs116794364
All − MR Egger
All − Inverse variance weighted
−1.0
−0.5
0.0
0.5
1.0
MR effect size for
'GCST90274819.csv' on 'outcome'
